# Supplementary material for: Complete genome sequences of Streptococcus pyogenes type strain reveal 100%-match between PacBio-solo and Illumina-Oxford Nanopore hybrid assemblies
Source: Sci Rep. 2020 Jul 15;10:11656. doi: 10.1038/s41598-020-68249-y (PMC7363880; doi:10.1038/s41598-020-68249-y)
Supplement: Supplementary file 1 — Supplementary Information [file 41598_2020_68249_MOESM1_ESM.docx]

# Supplementary information

# Complete genome sequences of *Streptococcus pyogenes* type strain reveal 100%-match between PacBio-solo and Illumina-Oxford Nanopore hybrid assemblies

**Francisco Salvà-Serra****^1,2,3,4,5,*^, Daniel Jaén-Luchoro****^1,2,3,4^, Hedvig E. Jakobsson^1,2,3,4^, Lucia Gonzales-Siles^1,2,3,4^, Roger Karlsson^1,2,3,4,6^, Antonio Busquets^5^, Margarita Gomila^5^, Antoni Bennasar-Figueras^5^, Julie E. Russell^7^, Mohammed Abbas Fazal^7^, Sarah Alexander^7^, Edward R. B. Moore^1,2,3,4^**

^1^Department of Infectious Diseases, Institute of Biomedicine, Sahlgrenska Academy, University of Gothenburg, Gothenburg, SE-413 46, Sweden; ^2^Culture Collection University of Gothenburg (CCUG), Sahlgrenska Academy, University of Gothenburg, Gothenburg, SE-413 46, Sweden; ^3^Department of Clinical Microbiology, Sahlgrenska University Hospital, Region Västra Götaland, Gothenburg, SE-413 46, Sweden; ^4^Centre for Antibiotic Resistance Research (CARe), University of Gothenburg, Gothenburg, SE-413 46, Sweden; ^5^Microbiology, Department of Biology, University of the Balearic Islands, Palma de Mallorca, E-07122, Spain; ^6^Nanoxis Consulting AB, Gothenburg, SE-400 16, Sweden; ^7^National Collection of Type Cultures (NCTC), Public Health England, London, NW9 5EQ, United Kingdom.

^*^Corresponding author: Francisco Salvà-Serra. Culture Collection University of Gothenburg (CCUG), Guldhedsgatan 10A, Gothenburg, SE-413 46, Sweden. Phone number: +46 737 572 402 / +34 666 397 284. E-mail address: [francisco.salva.serra@gu.se](mailto:francisco.salva.serra@gu.se)

**Supplementary Table 1.** Genes encoding putative virulence factors, identified using the Virulence Factors Database.

| **VFDB category** | **Gene** | **Locus tag** | **Phage region** | **Virulence factor** |
| --- | --- | --- | --- | --- |
| **Antiphagocytosis** | *emm* | DB248_RS09295 | - | M protein type 1 |
|  | *hasA* | DB248_RS09950 | - | Hyaluronic acid capsule |
|  | *hasB* | DB248_RS09955 | - | Hyaluronic acid capsule |
|  | *hasC* | DB248_RS09965 | - | Hyaluronic acid capsule |
|  | *sic* | DB248_RS09285 | - | SIC (streptococcal inhibitor of complement-mediated lysis) |
| **Exoenzymes** | *mf/spd* | DB248_RS09395 | - | DNaseB |
|  | *mf3/spd3* | DB248_RS06505 | SF130.4 | DNaseC |
|  | *hylA* | DB248_RS04240 | - | Hyaluronidase |
|  | *hylP* | DB248_RS03100 | SF130.1 | Hyaluronidase |
|  | *hylP* | DB248_RS04540 | SF130.2 | Hyaluronidase |
|  | *hylP* | DB248_RS06545 | SF130.4 | Hyaluronidase |
|  | *hylP* | DB248_RS07275 | SF130.5 | Hyaluronidase |
|  | *ideS/mac* | DB248_RS03795 | - | IgG-degrading enzyme |
| **Immune evasion** | *scpA* | DB248_RS09275 | - | C5a peptidase |
| **Plasminogen activator** | *ska* | DB248_RS09135 | - | Streptokinase |
| **Toxin** | *speA* | DB248_RS05660 | SF130.3 | Streptococcal pyrogenic exotoxin A |
|  | *speB* | DB248_RS09375 | - | Streptococcal pyrogenic exotoxin B |
|  | *speG* | DB248_RS01135 | - | Streptococcal pyrogenic exotoxin G |
|  | *speJ* | DB248_RS01990 | - | Streptococcal pyrogenic exotoxin J |
|  | *smeZ* | DB248_RS09220 | - | Streptococcal mitogenic exotoxin Z |
| **Adherence** | *cpa* | DB248_RS00770 | - | Pilus |
|  | *lepA* | DB248_RS00775 | - | Pilus |
|  | *fctA* | DB248_RS00780 | - | Pilus |
|  | *srtC1* | DB248_RS00785 | - | Pilus |
|  | *fctB* | DB248_RS00790 | - | Pilus |
|  | *fbaA* | DB248_RS09270 | - | FBPs (Fibronectin binding proteins) |
|  | *fbp54* | DB248_RS04160 | - | FBPs (Fibronectin binding proteins) |
|  | *lpb* | DB248_RS09265 | - | Laminin-binding protein |
| **Anti-proteolysis** | *grab* | DB248_RS06200 | - | GRAB (G-related α_2_-macroglobulin-binding protein) |
